# Determinants of reliability of self-reported height and weight and their impact on medication dosing: a cross-sectional study

**DOI:** 10.1136/bmjopen-2024-090020

**Published:** 2025-04-15

**Authors:** Markus Therre, Ingrid Kindermann, Sonja Maria Wedegärtner, Stephanie Groß, Igor Schwantke, Felix Mahfoud, Michael Böhm

**Affiliations:** 1 Department of Cardiology, Angiology and Intensive Care Medicine, Saarland University Hospital, Homburg, Germany; 2 Faculty of Medicine, Saarland University, Homburg, Germany; 3 Department of Psychosomatic Medicine, Federal German Pension Agency, Teltow, Germany; 4 Department of Cardiology, University Heart Center, University Hospital Basel, Basel, Switzerland; 5 Cardiovascular Research Institute Basel (CRIB), University Heart Center, University Hospital Basel, Basel, Switzerland; 6 HOMICAREM–Homburg Institute for CardioRenalMetabolic Medicine, Saarland University Hospital, Homburg, Germany

**Keywords:** GENERAL MEDICINE (see Internal Medicine), Body Mass Index, Cross-Sectional Studies

## Abstract

**Objective:**

Patient-reported anthropometric measures, such as height and weight, are frequently used in clinical practice but are susceptible to reporting biases. This study aims to investigate the determinants of reliability of patient-reported anthropometric measures in patients in cardiology and general practice and their impact on potential medication dosing.

**Design:**

Cross-sectional study.

**Setting and methods:**

730 patients were recruited at the Clinic of Cardiology, Angiology and Intensive Care Medicine of Saarland University Hospital and a general medicine practice from November 2015 to December 2018. We assessed self-reported height and weight and compared them to calibrated measures immediately afterwards. Weight and height (optional with medical history) were self-reported via questionnaire. Interviews were conducted by female or male nursing staff or physicians.

**Outcome measures:**

The main outcomes were the deviation between patients’ self-reported height and weight from objective calibrated measures, as well as the amount of misdosing of exemplary drugs based on this deviation.

**Results:**

The mean height (SD) of the participants (36% were patients) was 170.92 (9.34) cm. Patients significantly overestimated their height by 1.82 cm (range: −8.00 to 11.00 cm). Misreporting was best predicted by age, with older patients providing more height overestimations. The mean weight was 84.25 (17.41) kg and was significantly underestimated by 1.49 kg (range: −36.00 to 26.00 kg). Misreporting was best predicted by higher body mass index, cognitive impairment and a longer duration since the last weighing, and self-reporting by questionnaires was associated with a higher under-reporting of weight. Unlike females, male patients exhibited a more pronounced tendency to under-report their weight when responding to questionnaires compared with face-to-face interviews. Comparison of doses for low-molecular-weight heparin according to self-reported versus calibrated weight revealed potential underdosing and overdosing in 17% and 77% of all patients, respectively. For the cytostatic agent doxorubicin, for instance, underdosing and overdosing would have been applied in 40% and 43% of all patients, respectively.

**Conclusions and relevance:**

Self-reported height and weight are often invalid, especially in patients who are older and overweight. Misreporting can lead to inappropriate drug dosing. Calibrated measurement of height and weight remains part of good clinical practice, and if self-reporting is unavoidable, personal interviews should be preferred over questionnaires.

**Trial registration number:**

https://clinicaltrials.gov/study/NCT04321057

**STRENGTHS AND LIMITATIONS OF THIS STUDY**

- The cohort consisted of a real-world sample with anthropometric measures comparable to recent findings from the German National Cohort.
- Assessment of self-reported weight and height was operationalised by modality, as well as the sex and profession of the interviewer, to identify determinants of reliability.
- The potential amount of drug misdosing based on invalid self-reported anthropometric measures was calculated.
- The influence of the interviewer’s gender and profession on self-reports should be interpreted cautiously and replicated in a sample with equal distributions across all conditions.

## Introduction

 Patient’s height and weight are indispensable measures in clinical practice.^1 2^ Several medications, such as low-molecular-weight heparin, thrombolytic agents and glycoprotein IIb/IIIa inhibitors, are dosed according to weight.^3 4^ Other treatments, such as anticancer drugs, are administered based on body surface area (BSA), which is determined by both height and weight.^5^ Therefore, the inaccuracy of self-reported anthropometric measures can lead to incorrect drug dosing.^6^ Conditions like heart failure require regular weight monitoring, underlining the need for valid anthropometric measurements. In addition to time constraints in clinical practice,^4^ there are challenges to calibrated direct assessments, especially in intensive care settings,^7^ for patients who are wheelchair-dependent^8^ or bed-bound.^4^

Due to these limitations, self-reported data for height and weight are often used, despite the potential for bias.^9^ While some reports on the influence of gender, age, height and weight on the validity of self-reported measures are available,^10^,^12^ the influence of different modalities of acquisition has previously gained less attention (eg, questionnaire vs face-to-face interview).^10^ Therefore, our study aims to investigate the factors affecting the accuracy of self-reported height and weight and to assess the impact of survey methodology. To highlight the significance of these findings, we evaluated the impact of inaccurate self-reported measures on potential medication dosages.

## Methods

### Participants

**Figure 1:**
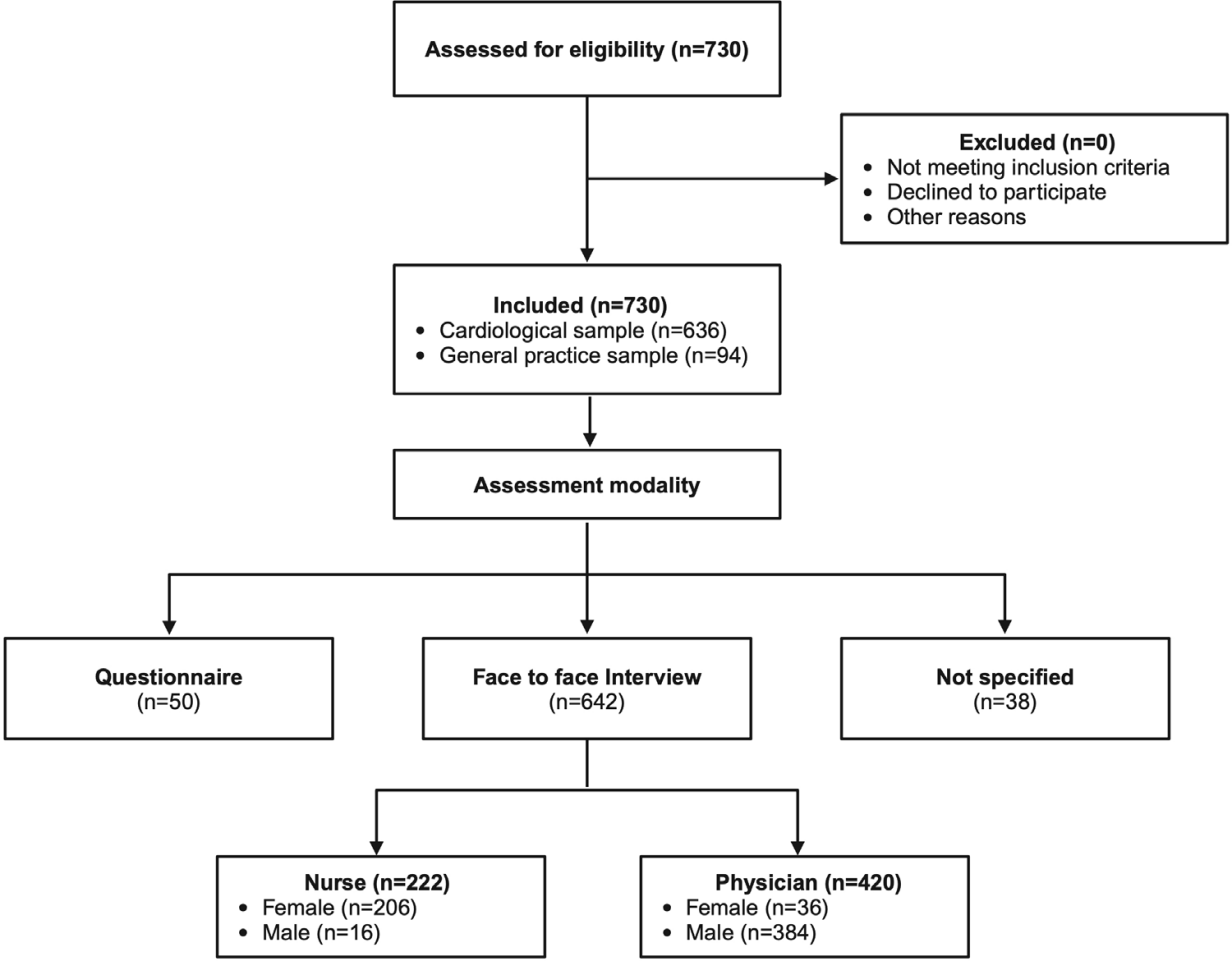
Consort flow diagram: assessment of patients and distribution to conditions.

Outpatients with cardiovascular disease from the Clinic of Cardiology, Angiology and Intensive Care Medicine at Saarland University Hospital and patients without a primary cardiovascular disease from a general practice were included in the study (for the consort flow diagram, see figure 1). Exclusion criteria included age<18 years, inability to independently fill in the questionnaire or measure height and weight and factors that likely impact these abilities, such as dementia, acute renal failure, cardiogenic shock, acute cardiac decompensation and severe anaemia (Hb<9 g/L). Ethical approval was obtained from the ethics committee of the Saarland Medical Association under the approval number 175/15. The study was conducted in accordance with the Declaration of Helsinki. All participants provided written informed consent.

### Measures

Height, self-reported height, height deviation between self-reported and calibrated measurements and the time point of the last self-assessed height (today/days ago/weeks ago/months ago/years ago) were assessed. Self-reported weight value, weight deviation between self-reported and calibrated measures and the time point of the last self-assessed weight (today/days ago/weeks ago/months ago/years ago) were also collected. Body mass index (BMI) and BSA were explored based on calibrated measures, self-reported height and weight and deviation between calibrated and self-reported measures. BMI was calculated and classified according to the WHO recommendations as underweight (<18.5 kg/m^2^), normal weight (18.5–24.9 kg/m^2^), preobesity (25.0–29.9 kg/m^2^), obesity class I (30.0–34.9 kg/m^2^), obesity class II (35.0–39.9 kg/m^2^) and obesity class III (>40 kg/m^2^).^13^ BSA was calculated according to Bois and Bois.^14^ The demographic data comprised age and gender for all participants. Furthermore, 249 patients (34%) answered a questionnaire containing additional information concerning living circumstances, college entrance qualification (no/yes) and employment status. Cognitive status was assessed in 538 patients (74%) using the German version of the Rapid Dementia Screening.^15^ Symptoms of general anxiety and depression were assessed using the German version of the Hospital Anxiety and Depression Scale.^16^ Quality of life was assessed using the German version of the Short Form 12.^17^ The medical status was recorded using a medical history sheet, which included medications taken, cardiovascular diagnoses and symptoms, left ventricular ejection fraction and selected laboratory values (for details, see online supplemental table 1).

### Data acquisition and assessment modalities

First, patients provided information about their current height and weight, the last time of measurement and the frequency of measurement, without knowing that their current height and weight would be assessed by the end of data acquisition. Self-reporting took place under different assessment conditions, which were operationalised by three variables: (1) modality (questionnaire or face-to-face interview), (2) sex of the interviewer (female or male) and (3) profession of the interviewer (nurse or physician), resulting in five conditions: questionnaire (n=50), interview by a female nurse (n=206), interview by a male nurse (n=16), interview by a female physician (n=36) and interview by a male physician (n=384). After completion of the questionnaire and medical assessment, a nurse conducted calibrated measurements. In 38 patients, the assessment modality was not recorded. Data from these 38 patients were excluded from the analysis regarding the impact of the interview condition.

### Data analysis

The statistical analysis was conducted with IBM SPSS Statistics, V.25 (IBM Corporation, 2017). The descriptive statistics show means and SD for metric variables and frequencies for categorical variables (table 1), conducted using analysis of variance (ANOVA) and Pearson’s χ²-test, respectively. When observed values for a metric variable in a χ² test were <5, Fisher’s exact test was applied. To assess the validity of self-reported anthropometric measures, we compared calibrated measures with self-reported measures using paired t-tests. We also compared the validity of self-report data between patients in cardiology and general practice. We performed ANOVA with planned contrasts. To determine the effect of theory-derived predictors on deviations between self-reported height and weight and calibrated measures, we tested significant correlations by running Pearson correlations between deviations and a set of predictor variables (anthropometric measures and condition, as well as demographic, medical, laboratory and psychological variables). Second, we used stepwise regression analyses to predict deviations with the predictors that showed significant correlations to determine the effect of each predictor on the deviation while controlling for the influence of the remaining ones. Finally, we calculated the potential amount of incorrect dosing based on invalid anthropometric measures and compared results for the face-to-face modality with the questionnaire modality using the χ² test. All presented P values were two-sided, and the *α*-level was set to 0.05.

**Table 1:** Study sample characteristics and group comparisons according to gender and patient subsamples (for full information, see online supplemental table 1)

Variable	Gender		P value	Subsample		P value	Total
	Women	Men		Cardiology	General practice		
N (%)	265 (36.3)	465 (63.7)	**<0.001**	636 (87.1)	94 (12.9)	**<0.001**	730
Demographic							
Gender (woman/man)	N/A	N/A	N/A	207 (32.5)/429 (67.5)	58 (61.7)/36 (38.3)	**<0.001**	
Age (SD), range	62.15 (15.56), 18–90	65.85 (13.34), 21–92	**0.001**	64.69 (14.08), 21 to 92	63.27 (15.67), 18–86	0.37	64.51 (14.29), 18–92
Anthropometric measuresm (SD), range							
Height (cm)	162.71 (6.75), 145 to 180	175.60 (7.12), 157 to 198	**<0.001**	171.60 (9.09), 146 to 198	166.31 (9.75), 145 to 193	**<0.001**	170.92 (9.34), 145 to 198
Self-reported height (cm)	164.62 (6.62), 148 to 190	177.36 (6.86), 160 to 198	**<0.001**	173.47 (8.79), 148 to 198	167.71 (9.86), 150 to 193	**<0.001**	172.73 (9.13), 148 to 198
Height deviation (cm)	1.91 (2.38), −6 to 10	1.76 (2.30), −8 to 11	0.40	1.87 (2.36), −8 to 11	1.41 (2.13), −6 to 10	0.71	1.81 (2.33), −8 to 11
Weight (kg)	76.08 (18.00), 43.70 to 153.00	88.93 (15.24), 58.90 to 150.00	**<0.001**	84.86 (17.16), 43.70 to 153.00	80.20 (18.70), 46.20 to 136.00	**0.015**	84.25 (17.41), 43.70 to 153.00
Self-assessed weight (kg)	74.50 (17.13), 44.00 to 150.00	87.48 (15.41), 60.00 to 170.00	**<0.001**	83.42 (16.97), 44.00 to 170.00	78.37 (18.31), 46.00 to 130.00	**0.008**	82.76 (17.22), 44.00 to 170.00
Weight deviation (kg)	1.58 (2.83), −5.90 to 26.00	1.45 (3.17), −36.00 to 22.00	0.58	1.45 (2.91), −36.00 to 26.00	1.83 (3.89), −4.40 to 22.00	0.26	1.49 (3.05), −36.00 to 26.00
BMI	28.73 (6.55), 16.65 to 53.40	28.84 (4.56), 15.75 to 44.07	0.79	28.79 (5.31), 15.75 to 52.94	28.85 (5.76), 18.48 to 53.40	0.93	28.80 (5.36), 15.75 to 53.40
Self-reported BMI	27.48 (6.07), 16.53 to 52.26	27.81 (4.60), 16.28 to 55.51	0.41	27.68 (5.13), 16.28 to 55.51	27.71 (5.55), 17.30 to 52.62	0.95	27.69 (5.18), 16.28 to 55.51
BMI deviation	−1.25 (1.44), −11.10 to 1.82	−1.03 (1.39), −9.99 to 15.50	**0.04**	−1.11 (1.37), −11.10 to 15.50	−1.13 (1.68), −9.99 to 1.96	0.89	−1.12 (1.41), −11.10 to 15.50
BSA (n=730)	1.81 (0.20), 1.39 to 2.52	2.05 (0.28), 1.59 to 2.67	**<0.001**	1.97 (0.21), 1.39 to 2.67	1.88 (0.24), 1.39 to 2.53	**<0.001**	1.96 (0.22), 1.39 to 2.67
Self-reported BSA	1.81 (0.19), 1.38 to 2.50	2.05 (0.18), 1.63 to 2.69	**<0.001**	1.97 (0.21), 1.42 to 2.69	1.87 (0.24), 1.38 to 2.54	**<0.001**	1.96 (0.22), 1.38 to 2.69
BSA deviation	−0.0003 (0.32), −0.23 to 0.08	0.0001 (0.03), −0.23 to 0.17	0.90	0.0010 (0.03), −0.23to 0.17	−0.0071 (0.04), −0.23 to 0.07	**0.03**	−0.0001 (0.03), −0.23 to 0.17

Significance of bold values, p≤0.05

BMI, body mass index; BSA, body surface area; N, Total number of individuals; N/A, not available.

### Patient and public involvement

Patients or the public were not involved in the design, conduct, reporting or dissemination plans of this research.

## Results

### Patients’ characteristics

The final sample consisted of 730 patients: 636 patients from the Clinic of Cardiology, Angiology and Intensive Care Medicine of Saarland University Hospital and 94 from general practice. The mean age was 64.5 (14.3) years, with 265 patients being female (36%). For descriptive statistics, see table 1, and for full information, see online supplemental table 1.

### Validity of self-reported anthropometric measures

#### Height

**Figure 2:**
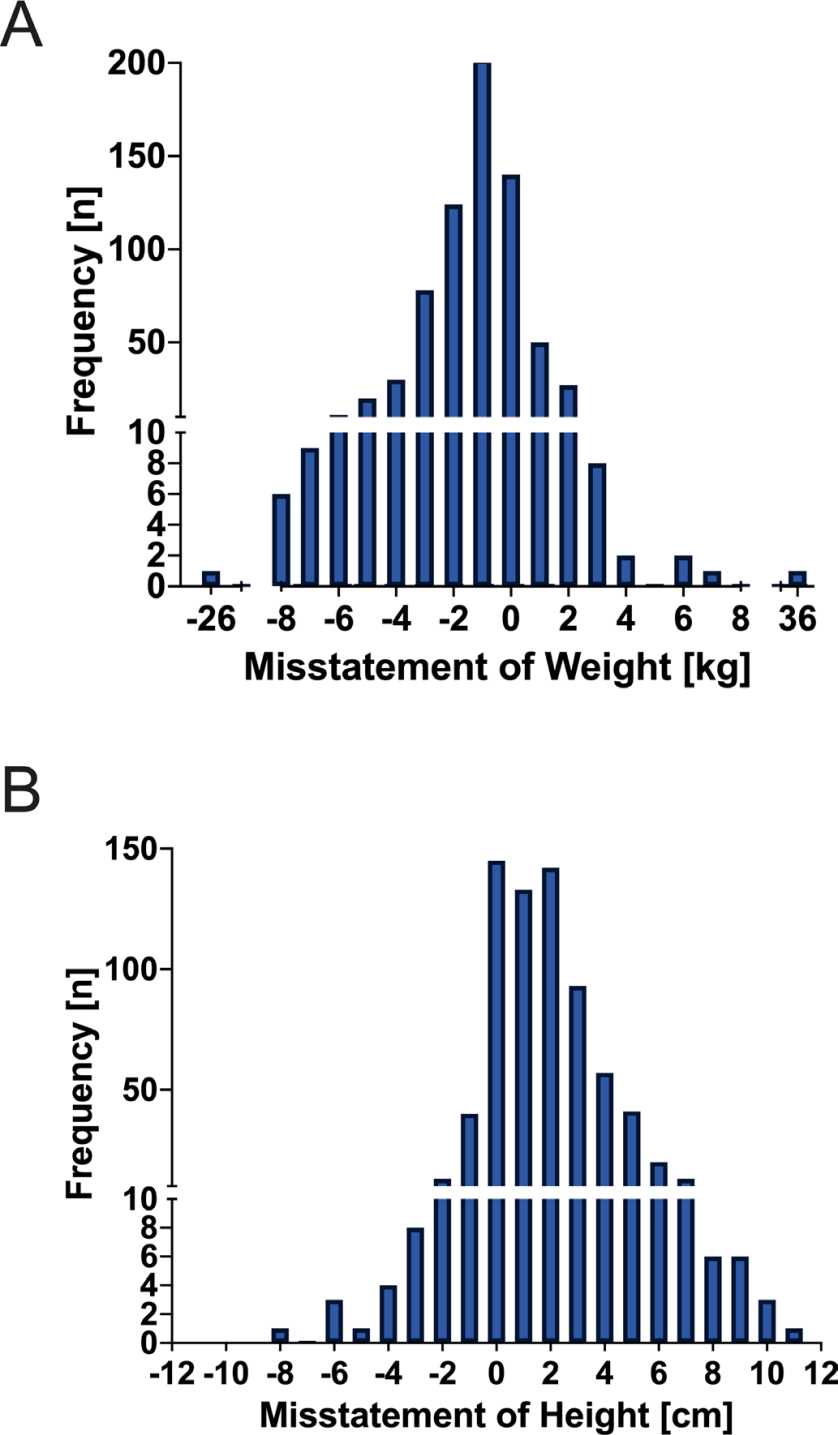
Frequency (n; ordinate) of misstatements in height (cm) and weight (kg) (abscissa).

**Figure 3:**
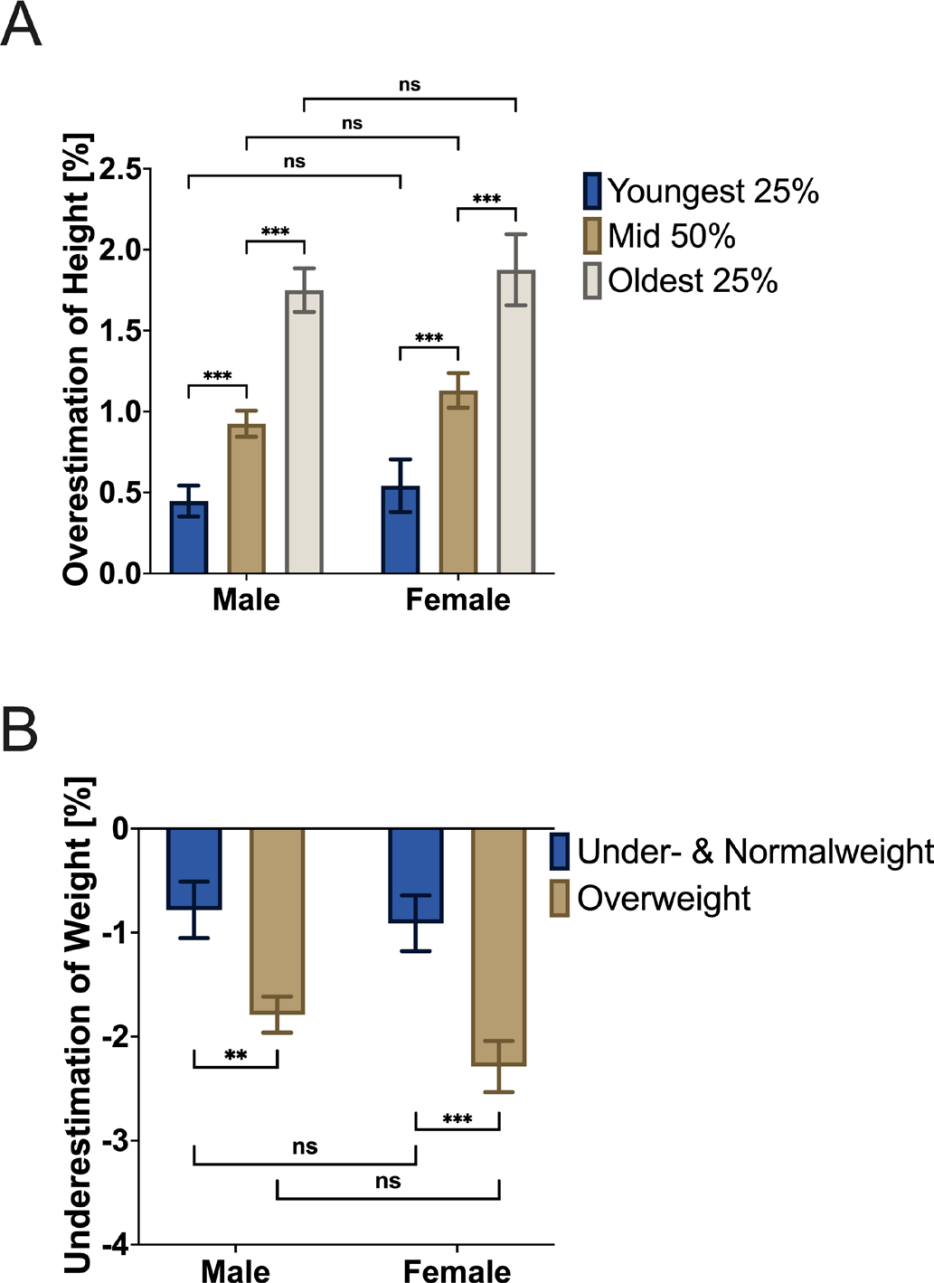
Overestimation of height (%) depending on gender and age (**A**) and underestimation of weight (%) depending on gender and body mass index (**B**). ns, not significant (p>0.05), **p≤ 0.01, ***p≤ 0.001.

The mean height (SD) of the participants was 170.92 (9.34) cm (female: 162.71 (6.75) cm and male: 175.60 (7.12) cm). A significant overestimation of self-reported height (172.73 (9.13) cm) compared with calibrated measures was observed, with a deviation of 1.82 (2.33) cm ((1.64 to 2.33); p<0.001 and range: −8.00 to 11.00 cm). 71% of patients over-reported their height (n=516), 19% reported their height correctly (n=141) and 10% under-reported their height (n=73) (see figure 2A). Overestimation was significantly more pronounced in patients below the median height compared with patients above the median (*t*(726)=5.80, p<0.001), which did not differ by sex. Analysis of height overestimation according to age quartiles and gender indicated that the oldest 25% of men and women overestimated their height significantly more than men and women in the middle 50% group. Furthermore, the middle-aged group overestimated their height significantly more than the youngest 25%. Men compared with women in all height quartiles did not differ in terms of height overestimation (see figure 3A).

#### Weight

The mean weight was 84.25 (17.41) kg (female: 76.08 (18.00) kg and male: 88.93 (15.24) kg). Self-reported weight of 82.77 (17.22) kg differed significantly from calibrated weight, with an underestimation of 1.49 (3.05) kg ((1.27 to 1.72); p<0.001 and range: −26 kg to 36 kg). 77% of patients under-reported their weight (n=559), 7% reported their weight correctly (n=50) and 17% over-reported their weight (n=121) (see figure 2B). When comparing the amount of underestimation by gender and BMI class, overweight men underestimated their weight significantly more than normal weight and underweight men. Overweight women also underestimated their weight significantly more than normal weight and underweight women (see figure 3B).

### Stepwise regressions for predictors of height and weight deviations

#### Height deviation

13 predictors correlated significantly with height deviation (see online supplemental table 2). To determine which of these correlates had a predictive value for height deviation over other predictors, we conducted a stepwise regression analysis. When predicting height deviation with these 13 values, a model including only age predicted the height deviation best and explained approximately 14% of the variance in height deviation (adjusted *R*²=0.137). Patients with higher age had higher overestimations in self-reported height compared with actual measured height.

#### Weight deviation

6 predictors correlated significantly with weight deviation (see online supplemental table 2). When predicting weight deviation with these 6 variables, a model including 4 of them predicted weight deviation best. These variables included calibrated BMI, cognitive impairment (none=1, slightly=2 and suspected dementia=3), condition (questionnaire=1/face-to-face=2) and duration since the last self-assessed measurement (1=today, 2=days, 3=weeks, 4=months and 5=years). Together they explained about 9% of the variance in weight deviation (adjusted *R*²=0.086). Patients with higher BMI, cognitive impairment, under the questionnaire condition and a longer duration since the last self-assessed measurement had a higher underestimation of self-reported weight compared with calibrated weight.

Correlations between modality and weight deviations in men (*r*=−0.161 (440); p*=*0.001) and women (*r*=0.029 (252); p=0.646) were tested against each other after applying Fisher's z-transformation to obtain a normal distribution. The analysis revealed a significantly higher weight deviation in men under the questionnaire modality (*z*=−2.411; p=0.008; see online supplemental figure 1).

### Analysis of height and weight deviations depending on assessment conditions

To account for the potential influence of actual height and weight, we compared the percentage deviations between self-reported and calibrated height and weight. One-way ANOVA, with values of height deviation, showed no statistically significant main effect of condition on height deviation. Contrast analysis, comparing each condition against one another, revealed significant mean differences between the female nurse and male doctor groups, with higher overestimations in the male doctor group.

One-way ANOVA using weight deviations revealed a statistically significant main effect of condition on weight deviation. Contrast analysis yielded three significant mean differences between conditions. Specifically, the questionnaire condition and the female nurse condition, the questionnaire condition and the male doctor condition and the questionnaire condition versus the overall interview conditions. Patients under the questionnaire condition had higher underestimations of weight than patients under the female nurse, male doctor and overall face-to-face conditions.

### Examples of invalid medication dosages based on invalid anthropometric measures

**Figure 4:**
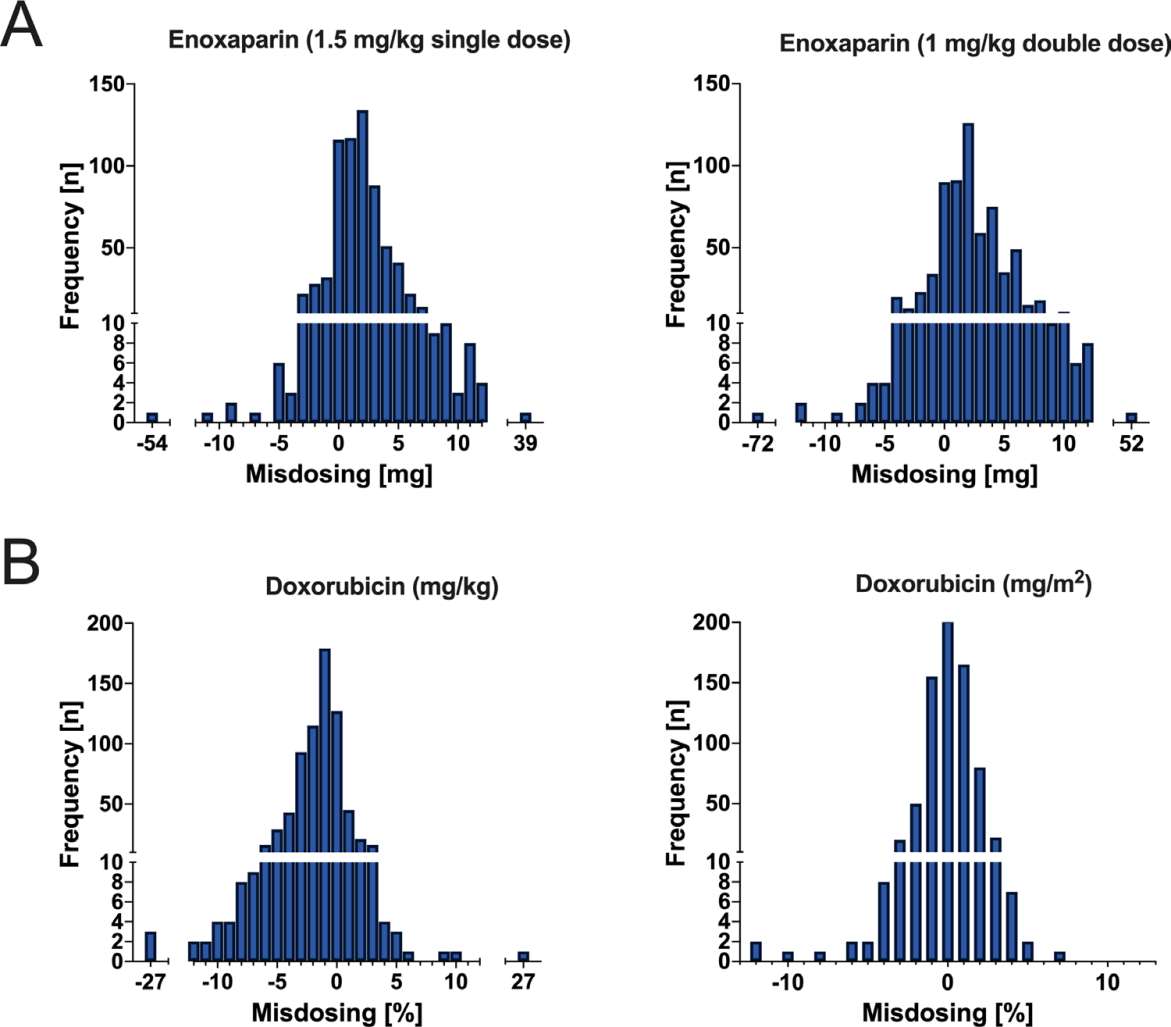
Frequency and amount of misdosing for enoxaparin (**A**) and doxorubicin (**B**).

In addition to fixed dosing, drugs are typically dosed based on body weight or BSA. One of the most commonly used medications that is dosed according to body weight is the anticoagulant enoxaparin, which has been shown to be one of the most frequently incorrectly dosed medications.^18 19^ Cytostatic drugs with a narrow therapeutic range are often dosed based on BSA in clinical practice. Doxorubicin, for example, has been approved for decades and is widely used as a first-line therapy for many solid and metastatic tumours. To evaluate the impact of inaccurate anthropometric measures on the potential dosage of selected medications, we compared dosages based on calibrated measures with dosages based on self-reported measures (see figure 4).

#### Low-molecular-weight heparin (enoxaparin)

For treatment of deep vein thrombosis and pulmonary embolism, enoxaparin is administered based on weight through subcutaneous injection either as a once-daily injection of 1.5 mg/kg or as two times per day injections of 1 mg/kg. In the case of 1.5 mg/kg administration, a comparison of dosage according to self-reported and calibrated weight revealed a potential underdosing in 121 patients (17%). Underdosing ranged from 0.15 mg to 54.00 mg, with a mean (SD) of 2.32 (5.06) mg. Overdosing would have been applied in 559 patients (77%), with a range from 0.15 mg to 39.00 mg and a mean of 3.43 (3.94) mg (see figure 4A). For two times per day, injections of 1 mg/kg drug administration would have also been too low in 121 patients (17%) and ranged from 0.10 mg to 36.00 mg, with a mean (SD) of 1.55 (3.38) mg for one dose and from 0.20 mg to 72.00 mg for two daily doses (mean (SD)=3.09 (6.75) mg). Overdosing would have been administered to 559 patients (77%), with a range from 0.10 mg to 26.00 mg and a mean (SD) of 2.29 (2.62) mg for one dose and from 0.20 mg to 52.00 mg, with a mean (SD) of 4.57 (5.25) mg for two daily doses (see figure 4A).

#### Cytostatic agent (doxorubicin)

We refer to the starting dose of a 3-week cycle in a single-agent regimen, given as a single dose according to BSA, as well as body weight. For doxorubicin monotherapy, the recommended standard starting dose per cycle in adults is 60–75 mg/m^2^ of BSA. For the calculation of misdosing, we used the lowest dose (60 mg/m^2^) and the highest dose (75 mg/m^2^). With respect to the lowest dose of 60 mg/m^2^, underdosing would have been applied in 290 patients (40%). Underdosing ranged from 0.41% to 12.17%, with a mean of 1.43% (1.46). With respect to the highest dose of 75 mg/m^2^, overdosing would have been applied in 317 patients (43%). Overdosing ranged from 0.37% to 6.75%, with a mean (SD) of 1.36% (0.94) (see figure 4B).

If the dosage is calculated based on body weight, 1.2–2.4 mg/kg is administered according to the Summary of Product Characteristics (SmPC) as a single dose every 3 weeks. Underdosing would have been applied in 558 patients (76%) and ranged from 0.09% to 27.37%, with a mean (SD) of 2.63% (2.85). Regarding the highest dose of 2.4 mg/kg, overdosing would have been applied to 119 patients (16%). Overdosing ranged from 0.10% to 26.87%, with a mean (SD) of 1.84% (2.80) (see figure 4B).

## Discussion

Notably, anthropometric measures in our sample are comparable to very recent findings from the German National Cohort.^20^ The BMI in our sample, with a mean of 28.80 (5.36) kg/m^2^, was comparable to another sample of 35 869 patients derived from general practitioners’ and internists’ offices.^21^

In this study, patients significantly underestimated their weight and significantly overestimated their height, which is in line with previous studies.^22 23^ Underestimation of weight was significantly more pronounced in overweight and obese patients compared with patients within the underweight and normal weight ranges according to BMI. Predictors for the underestimation of weight also included cognitive impairment, a longer duration since the last self-assessed measurement and self-reporting of weight via questionnaire. Exaggerating height, on the other hand, was more distinct in smaller people according to height quartiles. Notably, age was a predictor for the overestimation of height. These findings refer to the so-called ‘flat slope syndrome,’ the tendency to underestimate high values and overestimate low values.^24^

These observations include the social desirability bias^25^ and a lack of awareness of the actual weight or height.^26 27^ The social desirability bias describes the tendency to respond to sensitive questions according to perceived social norms rather than reality or truth. Accordingly, weight was more likely underestimated than overestimated, and underestimation was more pronounced in heavier people. These results are in line with previous studies that reported higher underestimations in women compared with men and younger women compared with older women.^10 28^ Height, on the other hand, was more likely over-reported than under-reported, and overestimation was more pronounced in persons below median height. Notably, in our study, gender was not associated with the number of misreports, neither for weight nor for height. However, the impact of social desirability bias on self-reports in our sample is further supported by the finding that self-reported weight measures were most accurate when obtained under face-to-face interview conditions and less accurate when surveyed by questionnaires. This extends the results of former studies, showing a higher degree of false reporting in telephone surveys compared with face-to-face surveys.^29 30^ A higher perceived level of accountability in the face-to-face situation might reduce misreports. Accordingly, self-reports of anthropometric measures are more accurate with the anticipation of being measured.^26^

The lack of awareness of one’s actual weight or height^27^ is likely to explain the observed misreporting. Age was predictive of higher overestimations in height. Since height is not frequently measured and tends to decrease with age, people might report a value taken at a younger age.^31^ Accordingly, the time since the last measurement of height was associated with higher deviations, and age was significantly correlated with lower calibrated measures of height. For weight, the time since the last measurement and cognitive impairment were predictive of misreports, further supporting a lack of awareness of current weight. An increase in awareness of one’s weight or height could lead to more accurate self-reports.^25^ However, patients with heart failure, for whom regular self-performed weighing is highly recommended for the titration of diuretics and, therefore, should generally have a high awareness of their weight, did not report their weight more accurately than others.

To provide context to the findings, we calculated the influence of patients’ self-reported weight and height on drug dosage. Potential underdosing of enoxaparin ranged up to 72.00 mg and overdosing up to 52.00 mg. A previous study reported high rates of incorrect low-molecular-weight heparin dosage in cardiovascular patients, affecting 49% of patients.^4^ Both underdosing and overdosing of enoxaparin can have harmful consequences. If the dosage is too low, patients may not experience complete dissolution of thrombosis or embolus. Conversely, the most dangerous side effect of overdosing on enoxaparin is excessive anticoagulation, leading to spontaneous bleeding, which can occur in various locations such as the gastrointestinal tract or brain. When it comes to doxorubicin, the risk of significant misdosing seems to be particularly high when dosing is based on body weight. In our sample, this led to a potential overdose of 10–27%. According to BSA, the highest potential overdose was 7%. While underdosing on doxorubicin leads to ineffective cancer treatment and disease progression, overdosing carries significant risks, most notably cardiotoxicity, severe damage to healthy tissues, organ failure and an increased risk of secondary cancers. Given that drug-related errors are a major factor associated with iatrogenic injury in patients,^32 33^ results are anyway far from being negligible.

### Limitations

The generalisability of our results might be limited by selection bias. Our sample consisted mainly of Caucasian men, with women making up 36% of the study population. Results regarding weight and height deviations between self-reported and calibrated measures are consistent with previous findings, diminishing concerns regarding generalisability. The impact of the interviewer’s gender and profession on self-reported anthropometric values should be interpreted cautiously and replicated in a sample where equal distribution is ensured across all conditions. Another important limitation of the study is that the calculation of potential incorrect drug dosages is purely hypothetical, which is helpful for exploring general trends or outcomes in controlled settings. However, these calculations likely oversimplify the complexities of real-world clinical practice, which range from patient and disease variability to pharmacokinetic and pharmacodynamic factors. These findings emphasise the importance of using real-world data, clinical expertise and personalised patient care when evaluating the effects of drug misdosing.

## Conclusions

**Figure 5:**
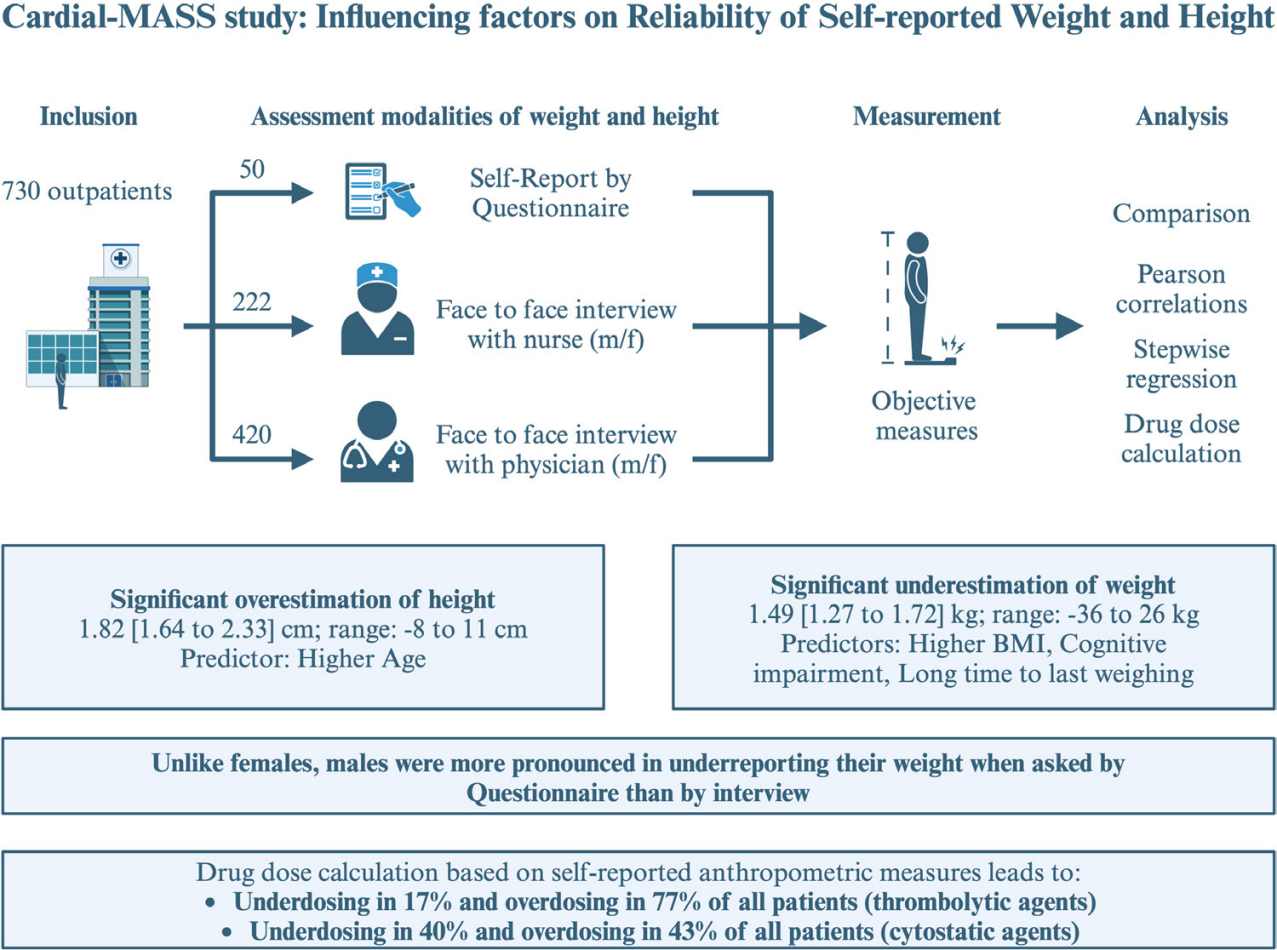
We included 730 outpatients, divided them into different assessment modalities of weight and height and compared these with objective measurements afterwards. We conducted Pearson correlations and stepwise regression analyses to identify predictor variables of misstatements. BMI, body mass index.

Anthropometric measures should be assessed using objective measurements, as self-reports are often inaccurate and can result in incorrect BMI classification and medication dosing. Factors such as age, cognitive impairment and the time since the last measurement, but not gender, were associated with misreporting. If self-reporting is inevitable, a face-to-face interview should be preferred over a questionnaire assessment. A concise summary can be found in figure 5.

## Supplementary material

10.1136/bmjopen-2024-090020 online supplemental file 1

## Data Availability

All data relevant to the study are included in the article or uploaded as supplementary information.
