# Supplementary material for: Determinants of reliability of self-reported height and weight and their impact on medication dosing: a cross-sectional study
Source: BMJ Open. 2025 Apr 15;15(4):e090020. doi: 10.1136/bmjopen-2024-090020 (PMC12001360; doi:10.1136/bmjopen-2024-090020)
Supplement: online supplemental file 1 [file bmjopen-15-4-s001.pdf]

Supplementary Data

Determinants of Reliability of Self-reported Height and Weight and their Impact on Medication Dosing

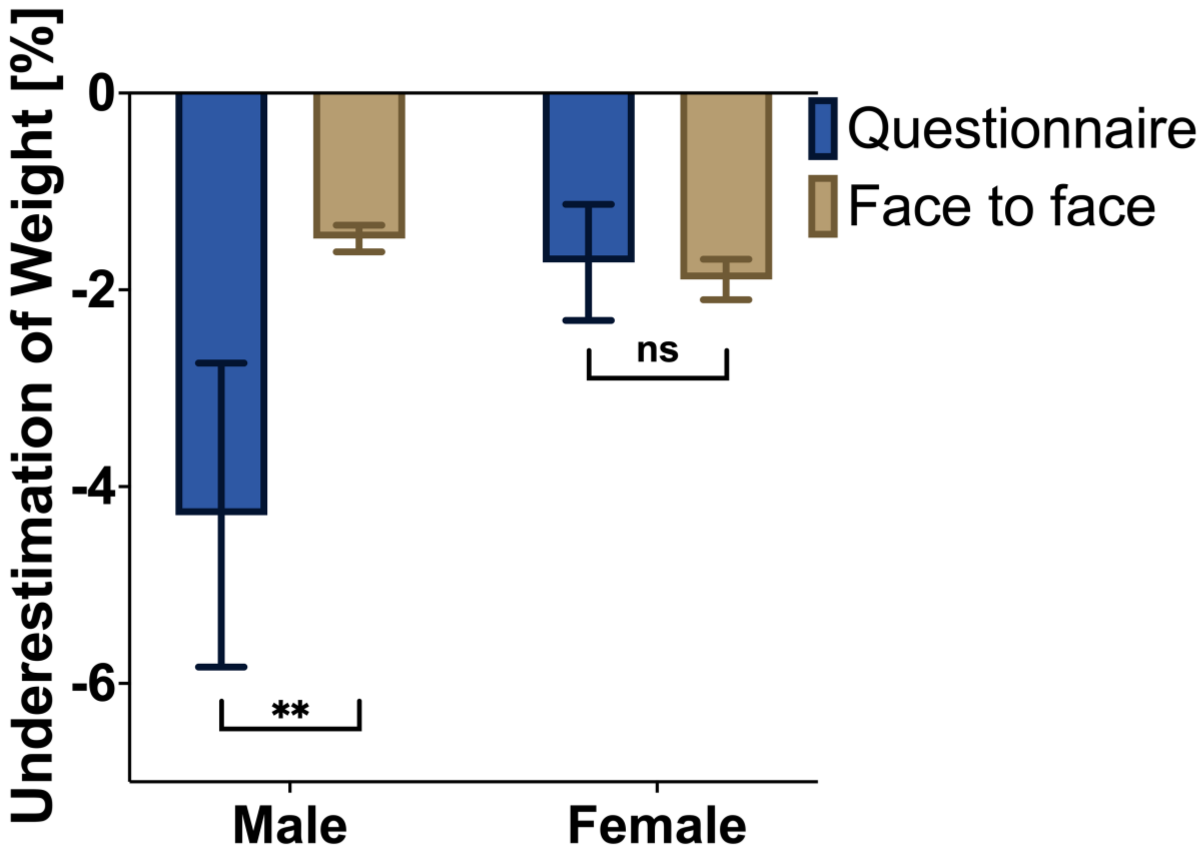

Supplement Figure 1: Amount of weight-underestimation in male and female (%; ordinate) in dependence of assessment modality (abscissa).

Supplement Table 1: Study sample characteristics and group comparisons according to gender and patient-subsamples.

	Gender			Sub-Sample			
**Variable**	**Women**	**Men**	***P***	**Cardiological**	**General Practice**	***P***	**Total**
N (%)	265 (36.3)	465 (63.7)	**<.001**	636 (87.1)	94 (12.9)	**<.001**	730
**Demographic**							
Gender (f/m)	N/A	N/A	N/A	207(32.5)/<br>429 (67.5)	58 (61.7)/<br>36 (38.3)	**<.001**	
Age (SD),<br>range	62.15 (15.56),<br>18-90	65.85 (13.34),<br>21-92	**.001**	64.69 (14.08),<br>21-92	63.27 (15.67),<br>18-86	.37	64.51 (14.29),<br>18-92
Living alone no/yes (%)<br>(n=247)	92 (70.2)/<br>39 (29.8)	98 (84.5)/<br>18 (15.5)	**.01**	121 (79.1)/<br>32 (20.9)	69 (73.4)/<br>25 (26.6)	.30	190 (76.9)/<br>57 (23.1)
Collegeantrance qualification no/yes<br>(n=247)	106 (80.9)/<br>25 (19.1)	87 (75.0)/<br>29 (25.0)	.26	106 (69.3)/<br>47 (30.7)	87 (92.6)/<br>7 (7.4)	**<.001**	193 (78.1)/<br>54 (21.9)
Retiered no/yes (%)<br>(n=247)	71 (54.2)/<br>60 (45.8)	28 (21.1)/<br>88 (75.9)	**<.001**	61 (39.9)/<br>92 (60.1)	38 (40.4)/<br>56 (59.6)	.93	99 (40.1)/<br>148 (59.9)
**Anthropometric measures**<br>m (SD), range							
Height in cm	162.71 (6.75),<br>145-180	175.60 (7.12),<br>157-198	**<.001**	171.60 (9.09),<br>146-198	166.31 (9.75),<br>145-193	**<.001**	170.92 (9.34),<br>145-198
Self-reported Height in cm	164.62 (6.62),<br>148-190	177.36 (6.86),<br>160-198	**<.001**	173.47 (8.79),<br>148-198	167.71 (9.86),<br>150-193	**<.001**	172.73 (9.13),<br>148-198

Height-deviation in kg	1.91 (2.38), -6-10	1.76 (2.30), -8-11	.40	1.87 (2.36), -8-11	1.41 (2.13), -6-10	.71	1.81 (2.33), -8-11
Weight in kg	76.08 (18.00), 43.70-153.00	88.93 (15.24), 58.90-150.00	**<.001**	84.86 (17.16), 43.70-153.00	80.20 (18.70), 46.20-136.00	**.015**	84.25 (17.41), 43.70-153.00
Self-assessed weight in kg	74.50 (17.13), 44.00-150.00	87.48 (15.41), 60.00-170.00	**<.001**	83.42 (16.97), 44.00-170.00	78.37 (18.31), 46.00-130.00	**.008**	82.76 (17.22), 44.00-170.00
Weight-deviation in kg	1.58 (2.83), -5.90-26.00	1.45 (3.17), -36.00-22.00	.58	1.45 (2.91), -36.00-26.00	1.83 (3.89), -4.40-22.00	.26	1.49 (3.05), -36.00-26.00
Timepoint of self-assessed weight (%) today/days ago/weeks/months ago/years ago (n=644)	72 (31.4)/ 61 (26.6)/ 60 (26.2)/ 22(9.6)/ 14 (6.1)	138 (33.3)/ 103 (24.8)/ 117 (28.2)/ 37 (8.9)/ 20 (4.8)	.90	198 (43)/ 151 (25.9)/ 153 (26.3)/ 50 (8.6)/ 30 (5.2)	12 (19.4)/ 13 (21)/ 24 (38.7)/ 9 (14.5)/ 4 (6.5)	**.04**	210 (32.6)/ 164 (25.5)/ 177 (27.5)/ 59 (9.2)/ 34 (5.3)
BMI	28.73 (6.55), 16.65-53.40	28.84 (4.56), 15.75-44.07	.79	28.79 (5.31), 15.75-52.94	28.85 (5.76), 18.48-53.40	.93	28.80 (5.36), 15.75-53.40
Self-reported BMI	27.48 (6.07), 16.53-52-26	27.81 (4.60), 16.28-55.51	.41	27.68 (5.13), 16.28-55.51	27.71 (5.55), 17.30-52.62	.95	27.69 (5.18), 16.28-55.51
BMI deviation	-1.25 (1.44), -11.10-1.82	-1.03 (1.39), -9.99-15.50	**.04**	-1.11 (1.37), -11.10-15.50	-1.13 (1.68), -9.99-1.96	.89	-1.12 (1.41), -11.10-15.50
BSA (n=730)	1.81 (0.20), 1.39-2.52	2.05 (0.28), 1.59-2.67	**<.001**	1.97 (0.21), 1.39-2.67	1.88 (0.24), 1.39-2.53	**<.001**	1.96 (0.22), 1.39-2.67
Self-reported BSA	1.81 (0.19), 1.38-2.50	2.05 (0.18), 1.63-2.69	**<.001**	1.97 (0.21), 1.42-2.69	1.87 (0.24), 1.38-2.54	**<.001**	1.96 (0.22), 1.38-2.69

BSA deviation	-0.0003 (0.32), -0.23-0.08	0.0001 (0.03) -0.23-0.17	.90	0.0010 (0.03), -0.23-0.17	-0.0071 (0.04), -0.23-0.07	**.03**	-0.0001 (0.03), -0.23-0.17
**Condition**							
Questionnaire/ face-to-face (n=692)	27 (10.7)/ 225 (89.3)	23 (5.2)/ 417 (94.8)	**.007**	30 (5)/ 568 (95)	20 (21.3) / 74 (78.7)	**<.001**	50 (7.2)/ 642 (92.8)
Profession of interviewer nurse/doctor (n=642)	97 (43.1)/ 128 (56.9)	125 (30)/ 292 (70)	**.001**	148 (26.1)/ 420 (73.9)	74 (100)/ 0	**<.001**	222 (34.6)/ 420 (65.4)
Gender of interviewer w/m (n=642)	108 (48)/ 117 (52)	135 (32.4)/ 282 (67.6)	**<.001**	169 (29.8)/ 399 (70.2)	74(100)/ 0	**<.001**	243 (37.9)/ 399 (62.1)
Gender of nurse f/m (n=222)	88 (90.7)/ 9 (9.8)	118 (94.4)/ 7 (5.6)	.29	132 (89.2)/ 16 (10.8)	74 (100)/ 0	**.003**	206 (92.8)/ 17 (7.2)
Gender of medical practitioner f/m (n=420)	20 (15.6)/ 108 (84.4)	17 (5.8)/ 275 (94.2)	**.001**	37 (8.8)/ 383 (91.2)	N/A	N/A	37 (8.8)/ 383 (91.2)
**Medical/laboratory variables**							
Number of medication, m (SD), range (n=660)	4.61 (3.72), 0-16	6.09 (3.60), 0-18	**<.001**	6.15 (3.55), 0-18	1.25 (0.93), 0-4	**<.001**	5.55 (3.71), 0-18
Hypertension n/y (n=564)	79 (36.2)/ 139 (63.8)	84 (24.3)/ 262 (75.7)	**.002**	129 (27.4)/ 341 (72.6)	34 (36.2)/ 60 (63.8)	.09	163 (28.9)/ 401 (71.1)
Diabetes mellitus n/y (n=564)	181 (83)/ 37 (17)	254 (73.4)/ 92 (26.6)	**.008**	365 (77.7)/ 105 (22.3)	70 (74.5)/ 24 (25.5)	.50	435 (77.1)/ 129 (22.9)
Hyperlipidemia n/y (n=564)	139 (63.8)/ 79 (36.2)	167 (48.3)/ 179 (51.7)	**<.001**	239 (50.9)/ 231 (49.1)	67 (71.3)/ 27 (28.7)	**<.001**	306 (41.9)/ 258 (45.7)

Coronary heart disease n/y (n=564)	169 (77.5)/ 49 (22.5)	157 (45.4)/ 189 (54.6)	**<.001**	262 (55.7)/ 208 (44.3)	64 (68.1)/ 30 (31.9)	**.03**	326 (57.8)/ 238 (42.2)
Atrial fibrillation n/y (n=564)	159 (72.9)/ 59 (27.1)	252 (72.8)/ 94 (27.2)	.98	329 (70)/ 141 (30)	82 (87.2)/ 12 (12.8)	**.001**	411 (72.9)/ 152 (27.1)
Myocardial infarction n/y (n=564)	198 (90.8)/ 20 (9.2)	276 (79.8)/ 70 (20.2)	**<.001**	382 (83.4)/ 78 (16.6)	82 (87.2)/ 12 (12.8)	.36	474 (84)/ 90 (12.3)
Reanimation n/y (n=564)	209 (95.9)/ 9 (4.1)	332 (96)/ 14 (4)	.96	453 (96.2)/ 18 (3.8)	89 (94.7)/ 5 (5.3)	.57	541 (95.9)/ 3.2 (4.1)
Apoplex n/y (n=564)	201 (92.2)/ 17 (7.8)	332 (96)/ 14 (4)	.06	442 (94)/ 28 (6)	91 (96.8)/ 3 (3.2)	.28	533 (94.5)/ 31 (6.5)
Heart failure n/y (n=564)	166 (76.1)/ 52 (23.9)	228 (65.9)/ 118 (34.1)	**.01**	314 (66.8)/ 156 (33.2)	80 (85.1)/ 14 (14.9)	**<.001**	394 (69.9)/ 170 (30.1)
Pacemaker n/y (n=564)	202 (92.7)/ 16 (7.3)	305 (88.2)/ 41 (11.8)	.08	416 (88.5)/ 54 (11.5)	91 (96.8)/ 3 (3.2)	**.02**	507 (89.9)/ 57 (10.1)
Implantable Cardioverter Defibrillator n/y (n=564)	202 (92.7)/ 16 (7.3)	290 (83.8)/ 56 (16.2)	**.002**	400 (85.1)/ 70 (14.9)	92 (97.9)/ 2 (2.1)	**<.001**	492 (87.2)/ 72 (12.8)
Previous hospital admission because of heart disease n/y (n=270)	76 (55.5)/ 61 (44.5)	42 (31.6)/ 91 (68.4)	**<.001**	46 (26.1)/ 130 (73.9)	72 (76.6)/ 22 (23.4)	**<.001**	118 (43.7)/ 152 (56.3)
Number of hospital admission because of heart disease (n=241)	1.30 (2.73), 0-10	2.16 (3.40), 0-10	**.03**	2.45 (3.56), 0-20	0.31 (0.85), 0-4	**<.001**	1.7 (3.01), 0-20
NYHA (I/II/III), N (%) (n=175)	39 (48.1)/ 41 (50.6)/ 1 (1.2)	29 (30.9)/ 61 (64.9)/ 4 (4.3)	**.04**	68 (38.9)/ 102 (58.3)/ 5 (2.9)	N/A	N/A	68 (38.9)/ 102 (58.3)/ 5 (2.9)

Ejection Fraction in %<br>m (SD), range (n=414)	56.55 (12.51),<br>15-81	52.14 (13.17),<br>13-18	**.001**	53.59 (13.11),<br>13-81	64.33 (5.51),<br>58-68	.16	53.66 (13.10),<br>13-81
**Laboratory Values**<br>m (SD), range							
Hemoglobin mg/dl (n=647)	13.51 (1.21),<br>9.60-17.90	14.78 (6.38),<br>8.70-140.00	**.003**	14.34 (5.44),<br>8.70-140.00	14.18 (1.31),<br>10.40-16.60	.82	14.33 (5.20),<br>8.70-140.00
NT-ProBNP pg/ml<br>(n=473)	996.05<br>(2393.94),<br>6.70-15200.00	802.93<br>(1751.40),<br>5.00-16435.99	.32	868.66 (1993.04),<br>5.00-16435.99	N/A	N/A	868.66<br>(1993.04),<br>5.00-16435.99
Calcium mg/dl<br>(n=637)	4.20 (0.45),<br>280-5.89	4.71 (6.96),<br>3.10-139.00	.28	4.54 (5.84),<br>2.80-139	4.36 (0.44),<br>3.61-5.89	.82	4.53 (5.60),<br>2.80-139
Creatinine mg/dl<br>(n=643)	1.31 (5.77),<br>0.48-88.00	1.24 (1.07),<br>0.60-21.00	.80	1.14 (0.93),<br>0.48-21.00	2.60 (11.73),<br>0.60-88.00	**.003**	1.26 (3.54),<br>0.48-88.00
GOT U/l<br>(n=634)	26.89 (15.06),<br>13.00-182.00	28.50 (10.66),<br>13.00-126.00	.12	28.09 (12.56),<br>13.00-182.00	26.21 (10.43),<br>15.00-75.00	.30	27.93 (12.40),<br>13.00-182.00
GPT U/l<br>(n=639)	25.44 (19.24),<br>6.00-168.00	30.01 (20.71),<br>5.00-311	**.006**	28.52 (20.63),<br>5.00-311.00	27.04 (16.64),<br>8.00-98.00	.60	28.39 (20.30),<br>5.00-311.00
Gamma-GT U/l<br>(n=637)	35.35 (37.37),<br>2.00-291.00	54.26 (71.35),<br>9.00-805.00	**<.001**	48.28 (64.00),<br>2.00-805.00	39.44 (35.30),<br>12.00-244.00	.31	47.49 (62.02),<br>2.00-805.00
Creatinine-GFR ml/min±SD<br>(n=605)	71.45 (21.04),<br>22.60-131	69.25 (21.53),<br>4.60-143	.23	21.51 (0.89),<br>4.60-143	65.65 (16.54),<br>31-98	.36	70.00 (21.37),<br>4.60-143.00
Bilirubin mg/dl	1.07 (4.71),	0.63 (0.37),	.27	0.81 (3.08),	0.35 (0.07),	.83	0.81 (3.07),

(n=242)	0.20-48	0.20-2.70		0.20-48.00	0.30-0.40		0.20-48.00
TSH µIU/ml (n=597)	1.73 (1.20), 0.01-10.02	1.82 (1.60), 0.01-17.66	.44	1.77 (1.45), 0.01-17.66	2.04 (1.69), 0.01-10.41	.25	1.79 (1.47), 0.01-17.66
Na mmol/l (n=630)	140.10 (3.03), 128.00-147.00	140.39 (3.49), 100.00-150.00	.29	140.38 (3.34), 100.00-150.00	139.19 (3.03), 132.00-146.00	**.02**	140.29 (3.33), 100.00-150.00
CRP mg/l±SD (n=592)	4.24 (8.17), 0.10-59.00-80.00	3.26 (6.13), 0.10-77.70	.10	3.67 (7.00), 0.10-77.70	1.51 (2.83), 0.10-12.10	.16	3.60 (6.90), 0.10-77.70
**Psychological measures**							
Cognitvely impaired (n/%) normal/ slightly/ severe (n=538)	180 (85.3)/ 22 (10.4)/ 9 (4.3)	288 (88.1)/ 24 (7.3)/ 15 (4.6)	.46	411 (92.6)/ 31 (7.0)/ 2 (0.5)	57 (60.6)/ 15 (16.0)/ 22 (23.4)	**<.001**	468 (87)/ 46 (8.6)/ 24 (4.5)
Anxiety symptoms normal/ slightly/ severe (n=247)	91 (69.5)/ 23 (17.6)/ 17 (13.0)	80 (69)/ 20 (17.2)/ 16 (13.8)	.98	103 (67.3)/ 32 (20.9)/ 18 (11.8)	68 (72.3)/ 11 (11.7)/ 15 (16.0)	.15	171 (69.2)/ 43 (17.4)/ 33 (13.4)
Depressive symptomatic normal/ slightly/ severe (n=247)	111 (84.7)/ 13 (9.9)/ 7 (5.3)	83 (71.6)/ 14 (12.1)/ 19 (16.4)	**.01**	121 (79.1)/ 17 (11.1)/ 16 (9.8)	73 (77.7)/ 10 (10.6)/ 11 (11.7)	.89	194 (78.5)/ 27 (10.9)/ 26 (10.5)

Abbreviations: N/A – not available, BMI – Body Mass Index, BSA – Body Surface Area, kg – Kilogram, cm – Centimeters, n/y – no/yes, GOT – Glutamate oxaloacetate transaminase, GPT – Glutamate pyruvate transaminase, Gamma-GT – Gamma-glutamyltransferase, GFR – Glomerular filtration rate, TSH – Thyroid Stimulating Hormone, Na – Sodium, CRP – C-reactive protein

Supplement Table 2: Correlations between deviations and demographic, medical/laboratory, and psychological predictors.

**Variable**	**Height-deviation**		**Weight-deviation**	
	**r**	***P***	**r**	***P***
**Anthropometric measures** (n=730)				
Actual height	-.213**	<.001	-.027	.47
Timepoint to last self-assessed measure (n=537)/(n=644)	.130**	.003	.094*	.02
Actual weight	-.070	.06	.156**	<.001
BMI	.049	.19	.206**	<.001
BSA	-.127**	.001	.113**	.002
**Demographic**				
Female gender (no/yes)*** (n=730)	.030	.40	.021	.58
Age (n=730)	.312**	<.001	-.015	.69
Living alone (no/yes)*** (n=247)	.031	.63	.010	.87
College entrance qualification (no/yes)*** (n=247)	-.048	.45	-.019	.77
Retired (no/yes)*** (n=247)	.099	.12	-.009	.89
Cardiological sample (no/yes)*** (n=730)	.067	.07	-.042	.26
**Condition**				
Face-to-face (no/yes)*** (n=692)	.029	.44	-.084*	.03
Interviewed by medical practitioner (no/yes)*** (n=642)	.108**	.006	-.040	.31
Male interviewer (no/yes) (=642)	.091*	.02	-.032	.43
Interview by male nurse (no/yes)*** (n=222)	.044	.51	.042	.53
Interview by male medical practitioner (no/yes)*** (n=420)	-.012	.81	-.015	.76
**Medical/laboratory variables**				
Number of medication (n=660)	.119**	.002	.006	.89

Hypertension (no/yes)*** (n=564)	.099*	.02	.077	.07
Diabetes mellitus (no/yes)*** (n=564)	-.014	.75	-.032	.45
Hyperlipidemia (no/yes)*** (n=564)	.023	.58	.050	.24
Coronary heart disease (no/yes)*** (n=564)	.152**	<.001	-.027	.52
Atrial fibrillation (no/yes)*** (n=564)	.115**	.006	.021	.62
Myocardial infarcton (no/yes)*** (n=564)	.073	.08	-.007	.87
Reanimation (no/yes)*** (n=564)	.002	.96	.005	.90
Apoplex (no/yes)*** (n=564)	.044	.30	.020	.63
Heart failure (no/yes)*** (n=564)	.033	.44	-.016	.71
Pacmaker (no/yes)*** (n=564)	.007	.86	.002	.96
Implantable Cardioverter Defibrillator (no/yes)*** (n=564)	.069	.10	-.021	.62
Privious hospital admission because of heart disease (no/yes)*** (n=270)	.089	.14	.056	.36
Number of previous hospital admission because of heart disease (n=241)	.079	.22	.043	.51
NYHA (n=175)	.078	.30	.014	.85
Ejection Fraction (n=414)	-.075	.13	.043	.38
**Laboratory Values**				
Hemoglobin mg/dl (n=647)	-.114**	.004	-.005	.91
NT-ProBNP pg/ml (n=473)	.095*	.04	.001	.99
Calium mg/dl (n=637)	.006	.87	-.016	.68
Creatinine mg/dl (n=643)	-.002	.96	.023	.56
GOT U/l	-.053	.18	-.019	.63

(n=634)				
GPT U/l (n=639)	-.052	.19	-.022	.58
Gamma-GT U/l (n=637)	-.024	.55	-.004	.92
Crea-GFR ml/min±SD (n=605)	-.201**	<.001	-.033	.42
Bilirubin mg/dl (n=242)	-.022	.74	-.048	.46
TSH µIU/ml (n=597)	.022	.58	.059	.15
Na mmol/l (n=630)	.018	.66	-.003	.95
CRP mg/l±SD (n=592)	.034	.41	.059	.15
**Psychological measures**				
Cognitvely impaired**** (n=538)	.015	.74	.121**	.005
Anxiety symptoms (n=247)	-.055	.39	.025	.70
Depressive symptomatic (n=247)	.040	.53	.042	.51

Abbreviations:BMI – Body Mass Index, BSA – Body Surface Area, NT-ProBNP – NTerminal Pro Brain Natriuretic Peptide, GOT – Glutamate oxaloacetate transaminase, GPT – Glutamate pyruvate transaminase, Gamma-GT – Gamma-glutamyltransferase, Crea-GFR – Creatinine-Glomerular filtration rate, TSH – Thyroid-stimulating hormone, Na – Sodium, CRP – C-reactive proteine

*Statistically significant on alpha .05

** statistically significant on alpha .01

***”no” was given the lower value in correlational analysis

****Reference values refer to 0=not impaired, 1= slightly impaired, 3=suspected dementia
