# Supplementary material for: Determinants of reliability of self-reported height and weight and their impact on medication dosing: a cross-sectional study
Source: BMJ Open. 2025 Apr 15;15(4):e090020. doi: 10.1136/bmjopen-2024-090020 (PMC12001360; doi:10.1136/bmjopen-2024-090020)
Supplement: Supplementary file 2 [file bmjopen-2024-090020.reviewer_comments.pdf]

PEER REVIEW HISTORY

BMJ Open publishes all reviews undertaken for accepted manuscripts. Reviewers are asked to complete a checklist review form and are provided with free text boxes to elaborate on their assessment. These free text comments are reproduced below.

ARTICLE DETAILS

Title (Provisional)

Determinants of reliability of self-reported height and weight and their impact on medication dosing: a cross-sectional study

Authors

Therre, Markus; Kindermann, Ingrid; Wedegärtner, Sonja Maria; Groß, Stephanie; Schwantke, Igor; Mahfoud, Felix; Böhm, Michael

---

VERSION 1 - REVIEW

**Reviewer**	**1**
**Name**	**Cloyd, Colleen P.**
**Affiliation**	**Department of Pharmacy, Nationwide Children's Hospital**
**Date**	**28-Aug-2024**
**COI**	**I have no competing interests to report.**

This paper addresses a common occurrence of self-reported height and weight at medical visits and nicely demonstrates how self-reported metrics can be inaccurate. Furthermore, the authors use this data to demonstrate how potential over and underdosing of medications could occur if only self-reported height and weight are used for dosing calculations.

This manuscript is very well-written. The objectives are clear and the language is succinct and effective. I have only a few minor suggestions to enhance this paper, should it be considered for acceptance.

1. In the discussion section, I'd appreciate a bit more speculation on the potential clinical outcomes of misdoing the medications you assessed. You chose important medications to highlight - all of which can have very deleterious effects if not dosed correctly. A bit more correlation here to potential clinical outcomes of over or underdosing these medications would give more emphasis to the need for measured heights and weights at each clinic visit/calculation of medication dosing.

2. Likewise, please add that you did not assess actual clinical outcomes of patients to your limitations section. The fact that the medication dosing section is all hypothetical should be overtly stated as a limitation. And perhaps as a future research question/outcome study.

**Reviewer**	**2**
**Name**	**Steadman, Kathryn**
**Affiliation**	**University of Queensland**
**Date**	**13-Oct-2024**
**COI**	**None**

This manuscript was a delight to review! This is an interesting and important article. The presentation of the figures is particularly informative. Congratulations to the authors on completing a very poignant study and preparing such a well written document. The English is a high standard throughout - the only sentence I noted could be improved is one of the boxes in the graphical abstract: "Unlike females, males were more pronounced in underreporting their weight when asked by questionnaire than by interview" would be more grammatically correct.

**Reviewer**	**3**
**Name**	**Munger, Mark**
**Affiliation**	**University of Utah College of Pharmacy, Pharmacotherapy**
**Date**	**06-Jan-2025**
**COI**	**None**

This manuscript reports 730 self-reported height and weight versus calibrated measures of the same anthropometric measures. For a simple study, the manuscript is excessively long, reports collecting data that are not reported in the manuscript (i.e., standardized questionnaires assessing anxiety, depression, QoL, and cognitive abilities) or assessed as reasons for discrepancy reporting between patient recollection and actual measurements, and does not add much to the literature in this field. The ability of patients to recall accurately their weight and height has been known for a number of years. The choice of the drugs that the authors used is not defended in the manuscript. Finally, there are number of grammatical errors throughout the manuscript that need changing.

VERSION 1 - AUTHOR RESPONSE

Reviewers' Comments to the Author:

Reviewer 1 (Dr. Colleen P. Cloyd, Department of Pharmacy, Nationwide Children's Hospital):

This paper addresses a common occurrence of self-reported height and weight at medical visits and nicely demonstrates how self-reported metrics can be inaccurate. Furthermore, the authors use this data to demonstrate how potential over and underdosing of medications could occur if only self-reported height and weight are used for dosing calculations. This manuscript is very well-written. The objectives are clear, and the language is succinct and effective. I have only a few minor suggestions to enhance this paper, should it be considered for acceptance.

1. In the discussion section, I'd appreciate a bit more speculation on the potential clinical outcomes of misdoing the medications you assessed. You chose important medications to highlight - all of which can have very deleterious effects if not dosed correctly. A bit more correlation here to potential clinical outcomes of over or underdosing these medications would give more emphasis to the need for measured heights and weights at each clinic visit/calculation of medication dosing.

Response 1: We thank the reviewer for her valuable suggestion. In order to enhance this point, we have provided a clearer explanation for why we selected these particular medications in the results section: “In addition to fixed dosing, drugs are typically dosed based on body weight or BSA. One of the most commonly used medications that is dosed according to body weight is the anticoagulant enoxaparin, which has been shown to be one of the most frequently incorrectly dosed medications [18,19]. Cytostatic drugs with a narrow therapeutic range are often dosed based on BSA in clinical practice. Doxorubicin, for example, has been approved for decades and is widely used as a first-line therapy for many solid and metastatic tumors. To evaluate the impact of inaccurate anthropometric measures on the potential dosage of selected medications, we compared dosage based on calibrated measures with dosages based on self-reported measures (see Figure 4).”

Additionally, we have speculated on the potential clinical outcomes that may result from misdosing of these drugs. The discussion has been revised as follows: “Both underdosing and overdosing of enoxaparin can have harmful consequences. If the dosage is too low, patients may not experience complete dissolution of thrombosis or embolus. Conversely, the most dangerous side effect of overdosing enoxaparin is excessive anticoagulation, leading to spontaneous bleeding, which can occur in

various locations such as the gastrointestinal tract or brain. When it comes to doxorubicin, the risk of significant misdosing seems to be particularly high when dosing is based on body weight. In our sample, this led to a potential overdosing of 10 to 27%. According to BSA the highest potential overdose was 7%. While underdosing of doxorubicin leads to ineffective cancer treatment and disease progression, overdosing carries significant risks, most notably cardiotoxicity, severe damage to healthy tissues, organ failure, and an increased risk of secondary cancers. Given that drug related errors are a major factor associated with iatrogenic injury in patients [32,33], results are anyway far from being negligible."

2. Likewise, please add that you did not assess actual clinical outcomes of patients to your limitations section. The fact that the medication dosing section is all hypothetical should be overtly stated as a limitation. And perhaps as a future research question/outcome study.

Response 2: Thank you for your comment. We completely agree that the medication dosing section is purely hypothetical. That's why we made sure to highlight this as an important limitation: "Another important limitation of the study is that the calculation of potential incorrect drug dosages is purely hypothetical, which is helpful for exploring general trends or outcomes in controlled settings. However, these calculations likely oversimplify the complexities of real-world clinical practice, which range from patient and disease variability to pharmacokinetic and pharmacodynamic factors. These findings emphasize the importance of utilizing real-world data, clinical expertise, and personalized patient care when evaluating the effects of drug misdosing."

Reviewer 2 (Dr. Kathryn Steadman, University of Queensland):

This manuscript was a delight to review! This is an interesting and important article. The presentation of the figures is particularly informative. Congratulations to the authors on completing a very poignant study and preparing such a well written document.

1. The English is a high standard throughout - the only sentence I noted could be improved is one of the boxes in the graphical abstract: "Unlike females, males were more pronounced in underreporting their weight when asked by questionnaire than by interview" would be more grammatically correct.

Response 1: We thank the reviewer for the improvement of the graphical abstract. The box in the graphical abstract was revised as follows: “Unlike females, males were more pronounced in underreporting their weight when asked by questionnaire than by interview.”

Reviewer 3 (Dr. Mark Munger, University of Utah College of Pharmacy):
This manuscript reports 730 self-reported height and weight versus calibrated measures of the same anthropometric measures.
1. For a simple study, the manuscript is excessively long, reports collecting data that are not reported in the manuscript (i.e., standardized questionnaires assessing anxiety, depression, QoL, and cognitive abilities) or assessed as reasons for discrepancy reporting between patient recollection and actual measurements, and does not add much to the literature in this field. The ability of patients to recall accurately their weight and height has been known for a number of years.

Response 1: We would like to express our gratitude to the reviewer for this valuable feedback. We acknowledge that the methods section is excessively long, particularly in relation to data used solely for the stepwise regression analysis or found only in a supplementary table. As a result, we have significantly condensed the methods and results sections in order to enhance the readability of the manuscript.
We totally agree that it is widely recognized that patients often inaccurately report their height and weight. However, this study specifically examines predictors of these misstatements, with a focus on assessment modalities to improve clinical practice. We have revised this part of the introduction section to clarify the objectives of our work: “While some reports on the influence of gender, age, height, and weight on the validity of self-reported measures are available [10–12], the influence of different modalities of acquisition has previously gained less attention (e.g., questionnaire vs. versus face-to-face interview) [10]. Therefore, our study aimed to investigate the factors affecting the accuracy of self-reported height and weight and expanded the study to examine the effects to assess the impact of survey methodology.”

2. The choice of the drugs that the authors used is not defended in the manuscript.

Response 2: We agree with the reviewer that our explanation of the drugs used for calculation was not clear enough. As mentioned above (Response to Reviewer 1), we have provided a more detailed explanation for why we selected these particular medications in the results section: “In addition to fixed dosing, drugs are typically dosed based on body weight or BSA. One of the most commonly used medications that is dosed according to body weight is the anticoagulant enoxaparin, which has been shown to be one of the most frequently incorrectly dosed medications [18,19]. Cytostatic drugs with a narrow therapeutic range are often dosed based on BSA in clinical practice. Doxorubicin, for example, has been approved for decades and is widely used as a first-line therapy for many solid and metastatic tumors. To evaluate the impact of inaccurate anthropometric measures on the potential dosage of selected medications, we compared dosage based on calibrated measures with dosages based on self-reported measures (see Figure 4).”
Furthermore, we have expanded on the potential clinical implications of misdosing these drugs in the discussion section: “Both underdosing and overdosing of enoxaparin can have harmful consequences. If the dosage is too low, patients may not experience complete dissolution of thrombosis or embolus. Conversely, the most dangerous side effect of overdosing enoxaparin is excessive anticoagulation, leading to spontaneous bleeding, which can occur in various locations such as the gastrointestinal tract or brain. When it comes to doxorubicin, the risk of significant misdosing seems to be particularly high when dosing is based on body weight. In our sample, this led to a potential overdosing of 10 to 27%. According to BSA the highest potential overdose was 7%. While underdosing of doxorubicin leads to ineffective cancer treatment and disease progression, overdosing carries significant risks, most notably cardiotoxicity, severe damage to healthy tissues, organ failure, and an increased risk of secondary cancers. Given that drug related errors are a major factor associated with iatrogenic injury in patients [32,33], results are anyway far from being negligible.”

3. Finally, there are number of grammatical errors throughout the manuscript that need changing.

Response 3: Thank you for bringing this to our attention. We consulted with language editing and were able to correct several additional grammatical errors. All individual changes can be tracked in the “tracked changes manuscript”.
